# A study to compare the efficacy of polyether ether ketone rod device with titanium devices in posterior spinal fusion in a canine model

**DOI:** 10.1186/s13018-017-0543-x

**Published:** 2017-03-09

**Authors:** Nanxiang Wang, Huanxin Xie, Chunyang Xi, Han Zhang, Jinglong Yan

**Affiliations:** Department of Orthopaedics, The Second Affiliated Hospital of Harbin Medical University, Harbin, 150081 China

**Keywords:** PEEK devices, Titanium rods, Paraspinal muscle-pediculated bone flaps, Posterior lumbar fusion, Canine models

## Abstract

**Background:**

The benefits of posterior lumbar fusion surgery with orthotopic paraspinal muscle-pediculated bone flaps are well established. However, the problem of non-union due to mechanical support is not completely resolved. The aim of the study was to compare the efficacy of polyether ether ketone (PEEK) rod device with conventional titanium devices in the posterior lumbar fusion surgery with orthotopic paraspinal muscle-pediculated bone flaps.

**Methods:**

This was a randomized controlled study with an experimental animal model. Thirty-two mongrel dogs were randomly divided into two groups—control group (*n* = 16), which received the titanium device and the treatment group (*n* = 16), which received PEEK rods. The animals were sacrificed 8 or 16 weeks after surgery. Lumbar spines of dogs in both groups were removed, harvested, and assessed for radiographic, biomechanical, and histological changes.

**Results:**

Results in the current study indicated that there was no significant difference in the lumbar spine of the control and treatment groups in terms of radiographic, manual palpation, and gross examination. However, certain parameters of biomechanical testing showed significant differences (*p* < 0.05) in stiffness and displacement, revealing a better fusion (treatment group showed decreased stiffness with decreased displacement) of the bone graft. Similarly, the histological analysis also revealed a significant fusion mass in both treatment and control groups (*p* < 0.05).

**Conclusions:**

These findings revealed that fixation using PEEK connecting rod could improve the union of the bone graft in the posterior lumbar spine fusion surgery compared with that of the titanium rod fixation.

**Electronic supplementary material:**

The online version of this article (doi:10.1186/s13018-017-0543-x) contains supplementary material, which is available to authorized users.

## Background

Lumbar degenerative disease is a common disease that occurs along in any area of the spine [1]. Surgical intervention (arthrodesis) has been considered as a treatment of choice for the lumbar degenerative disease. Currently, pedicle screw instrumentation is a preferred treatment for the spinal fusion back surgery as it leads to rapid stabilization and considerable improvement in the fusion process [2–5]. However, many patients during follow-up after the initial surgical intervention presented with persistent symptoms, progressive underlying degenerative disease, or other new symptoms, which required further surgical interventions. This pathology is known as “adjacent segment disease (ASD)”, in which the incidence rate was estimated to be 30 to 100% from the first year to the tenth year and occurs with a rate of 35 to 45% [6–8]. Several studies have raised issues regarding the use of rigid fusion devices that are made up of titanium rods, which presents increased stress on the nearby joints, vertebral discs, hypertrophy of facets, the appearance of osteophytes, stenosis at the lumbosacral region, abnormal movement, etc. In contrast to the above findings, dynamic stabilizing devices, which were made up of “compliant” material like polyether ether ketone (PEEK), reduce the risk of arthrodesis-induced complication of ASDs [9, 10].

PEEK is a synthetic biocompatible polymer with several advantages over titanium, such as stability maintenance, which showed stability loss with titanium rods after fatigue testing. In addition, the chance of loosening and breakage is comparatively higher in the case of titanium rods than PEEK rods. Furthermore, being a radiotransparent material, PEEK reduces the artifacts during radiological investigations [1, 9, 10]. The rigidity of titanium rods increases stress at the bone-screw interface, which increases the risk of fracture at that level. While, PEEK imposes less stress on the bone-screw interface due to its less rigid nature and thus decreases the incidence of degenerative diseases, fracture of pedicles, and avulsion of bone from the device [11].

PEEK, in addition to providing strength, also renders a certain degree of elasticity in maintaining the vertebra at the site of fusion in a state of micro-motion and therefore generates a micro-pressure on osteocytes. According to the Wolff law, the micro-pressure induced by the micro-motion could promote the union of the bone graft. However, whether this hypothesis could be verified in the pre-established modified bone grafting method or could interact with each other to further promote the union of the bone graft is still unclear. In this study, we have also modified the bone grafting method of operation and developed a new posterior lumbar spine bone grafting method using in situ paraspinal muscle-bone flaps, which was discussed previously [12].

In the present study, the effects of PEEK rod (dynamic fixation) were compared with that of titanium rod (rigid rod device) fixation on a canine model with posterior lumbar fusion along with orthotopic paraspinal muscle-pediculated bone flaps.

## Methods

### Study design

The study was conducted with the prior approval from Institutional Animal Ethics Committee. Thirty-two mongrel dogs with a mean body weight of 18.5 ± 1.0 kg and a mean age of 23 ± 3 months were selected randomly and divided into the control group, which received titanium rods and test group, which received PEEK rods.

### Surgical technique

The dogs were anesthetized by intravenous injection of barbanylum (60 mg/kg) and were endotracheally intubated. Each animal was given preoperative antibiotic prophylaxis (penicillin G 2.4 million units intravenous). After placing them in a prone position, the animals were shaved, disinfected with povidone-iodine, and prepared and draped, in a sterile manner.

In the control group, a midline incision was made from L5–L6 segment, then the paraspinal muscles were stripped subperiosteally from the spinous processes and laminae (on both sides) until superior articular processes (SAPs) were seen. The SAPs of L6 and pars interarticularis (PIs) of L5 were split vertically by sharp osteotome on both sides. Spinal arthrodesis was achieved using a WEGO/GB1Z Internal Fixator (Wego Ortho, Inc, Weihai, People’s Republic of China) that consists of four 4-mm diameter and 20-mm-long titanium pedicle screws which were placed in L5 and L6 bilaterally and were connected with two 3-mm diameter titanium rods. Bone graft was obtained through the same skin incision but through a different fascial approach. Autologous corticocancellous from both iliac crests was cleaned, trimmed, and morselized with a rongeur and was placed on the graft bed [11]. The bone graft was placed between the split medialis and lateral bone walls under internal fixation system. Autologous tricortical iliac bone (5 g) was equally distributed to the bilaterally decorticated arthrodesis sites at the appropriate lumbar level (2.5 g per side).

In the test group, spinal arthrodesis was achieved using 3.0 mm diameter PEEK rod replacing the titanium rod in WEGO/GB1Z Internal Fixator (3 mm). All other operation steps were same as carried out in the control group (Additional file 1: Figure S1 and Additional file 2: Figure S2).

### Radiographic assessment

All animals were sacrificed with an overdose of barbanylum at 8 or 16 weeks after surgery, and the spinal columns of each animal in both the groups were harvested, including segments L1–L7 along with all ligamentous structures. Following removal of the metal implants, anteroposterior radiographs and computed tomography (CT) scans were taken of the spinal specimens. Radiograph plates and three-dimensional reconstruction of CT of corresponding fused segments were evaluated together by two independent radiologists who were not aware of the types of treatment.

### Manual palpation and biomechanical testing

Soft tissues were removed from the operative segments after the radiographic analysis. The magnitude of flexion-extension motion of the segments analyzed by manual palpation was used to assess the fusion status. After that, each specimen was disarticulated into the L5–L6 functional spinal unit for biomechanical testing. Stainless steel screws were used to anchor the cephalad and caudal vertebrae of each functional spinal unit, and each segment was secured in the metal fixtures with polyester resin. This biomechanical testing included analysis of the motion in six directions, namely, flexion, extension, right and left bending, and right and left axial rotation. A custom 4-point bending flexural test device was used to test the flexion, extension, right, and left bending. All biomechanical tests were performed using an MTS 858 Mini Bionix testing device (MTS Systems, Minneapolis, MN, USA). All specimens were kept moist throughout the experiment (during both preparatory phase and testing phase) by physiologic saline solution [11].

### Histologic analysis

The specimens were harvested and then were fixed by immersing in 10% neutral-buffered formalin solution for at least 24 h. Ten percent of nitric acid was used for decalcification. Paraffin-embedded tissues were sectioned (thickness, 5 μm) and stained with hematoxylin and eosin (H&E). Five sequential sagittal sections of each specimen were examined. For each histologic section, three random ×10 fields were evaluated and scored. The quality of each section was fusion graded by assigning a histologic score from 0 to 7 [13]. The fusion mass was considered to have been fused when the maximum Emery tissue scale score was greater than or equal to 6.

### Statistical analysis

The SPSS 18.0 software package (SPSS Inc., Chicago, IL) was used for statistical analysis. Mean ± standard deviation was used to present the normal distribution of the data, and the median (range) was used to present the abnormal distribution. The radiographic, mechanical (by means of manual palpation), mechanical stiffness, and displacement were analyzed by *t* test or Wilcoxon rank-sum test according to the data normal distribution. Histologic data was divided into two groups according to the score ≥6 or not. Chi-square test was used to analyze the histologic results. Significance was defined as *p* < 0.05.

## Results

### Surgery results

All the animals survived the surgical procedure and were ambulated within 48 h of surgery. The average operative time was 130.2 ± 12.6 min in the treatment group and 135.6 ± 13.0 min in the control group (*p* > 0.05). There was no statistically significant differences observed in terms of intraoperative bleeding between the control and treatment group (*p* > 0.05).

### Radiographic evaluation and manual palpation

Radiographic evaluation performed at 8 and 16 weeks after surgery did not show any significant differences between the control and treatment groups (Table 1, Additional file 3: Figure S3 and Additional file 4: Figure S4). Manual palpation which was similar to that of radiographic evaluation failed to show any significant differences between both the groups (Table 2, Additional file 5: Figure S5).

**Table 1:** Radiographic evaluation—comparison between control and treatment groups at different time intervals

Scores at different time intervals	Total number of animals (missing)	Control group	Treatment group	*p* value
8-week score	8(0)	2.88 ± 0.96	2.94 ± 0.93	0.853
16-week score^a^	8(0)	4(3, 4)	4(3, 4)	0.898

^a^Means non-abnormal distribution and presented as median(range), analyzed using Wilcoxon rank-sum test

**Table 2:** Manual palpation and gross examination—comparison between control and treatment groups at different time intervals

Scores at different time intervals	Total number of animals (missing)	Control group	Treatment group	*p* value
8-week score	8(0)	1.13 ± 0.83	1.25 ± 0.71	0.751
16-week score^a^	8(0)	2(1, 2)	2(1, 2)	0.295

^a^Means non-abnormal distribution and presented as median(range), analyzed using Wilcoxon rank-sum test

### Biomechanical analysis

Some of the parameters of biomechanical analysis showed significant differences between the control and treatment groups.

At 8 weeks post-operation, the treatment group showed significantly increased stiffness in rotations than the control group (both the left and right; *p* < 0.01, Table 3a). But the biomechanical analysis in terms of displacement, lateroflexion in both directions and rotation showed less displacement in the treatment group compared to control (all *p* < 0.01, Table 3b).

**Table 3:** Biomechanical analysis in terms of stiffness—comparison between control and treatment group at different time intervals

Mechanical analysis: stiffness	8 weeks		16 weeks	
	Control group (*n* = 8)	Treatment group (*n* = 8)	Control group (*n* = 8)	Treatment group (*n* = 8)
Anteflexion (N/mm)	206.16 ± 19.01	205.61 ± 20.38	293.30 ± 9.24	307.66 ± 14.84^#^
Dorsiflexion (N/mm)	202.04 ± 21.76	202.96 ± 20.98	275.93 ± 11.50	278.91 ± 18.60
Left lateroflexion (N/mm)	178.95 ± 16.19	194.96 ± 19.72	287.24 ± 12.50	302.45 ± 15.12^#^
Right lateroflexion(N/mm)	179.74 ± 17.32	193.53 ± 16.00	273.48 ± 16.60	295.01 ± 16.13^#^
Left rotation (N/deg)	2.10 ± 0.26	2.59 ± 0.26*	3.67 ± 0.12	4.17 ± 0.19*
Right rotation (N/deg)	2.24 ± 0.12	2.61 ± 0.16*	4.07 ± 0.22	4.59 ± 0.19*

^#^ *p* < 0.05, *p < 0.01

At 16 weeks post-operation, the treatment group showed significantly increased stiffness in all the five directions except dorsiflexion in comparison to the control group (Table 3a). It also showed significantly reduced displacement in all the five directions except dorsiflexion compared with that of the control group (Table 3b, Table 4).

**Table 4:** Biomechanical analysis in terms of displacement—comparison between control and treatment group at different time intervals

Mechanical analysis: displacement	8 weeks		16 weeks	
	Control group (*n* = 8)	Treatment group (*n* = 8)	Control group (*n* = 8)	Treatment group (*n* = 8)
Anteflexion (N/mm)	0.51 ± 0.04	0.49 ± 0.03	0.35 ± 0.02	0.29 ± 0.02*
Dorsiflexion (N/mm)	0.51 ± 0.05	0.52 ± 0.04	0.38 ± 0.02	0.39 ± 0.03
Left lateroflexion (N/mm)	0.59 ± 0.05	0.51 ± 0.04*	0.36 ± 0.04	0.27 ± 0.04*
Right lateroflexion(N/mm)	0.62 ± 0.04	0.54 ± 0.05*	0.38 ± 0.03	0.29 ± 0.04*
Left rotation (N/deg)	2.14 ± 0.34	1.58 ± 0.26*	1.15 ± 0.21	0.64 ± 0.21*
Right rotation (N/deg)	1.99 ± 0.26	1.47 ± 0.24*	1.02 ± 0.26	0.62 ± 0.18*

*p < 0.01

### Analysis of histology

At 8 weeks, 18 of the 40 fusion masses (45%) in the control group were graded as 6 or greater, whereas 26 of the 40 fusion masses (65%) in the treatment group were graded as 6 or greater (*p* = 0.072). At 16 weeks, 38 of the 40 fusion masses (95%) in the treatment group were graded as 6 or greater, whereas 30 of the 40 fusion masses (75%) in control group were graded as 6 or greater (*p* = 0.012, Table 5).

**Table 5:** Histological analysis of control and treatment group at different time interval

	Score								*p* values
	0	1	2	3	4	5	6	7	
Control group (8 weeks)	0	0	2	5	6	9	8	10	0.072^a^
Treatment group (8 weeks)	0	0	1	1	2	10	12	14	
Control group (16 weeks)	0	0	0	1	3	6	14	16	0.012^a^
Treatment group (16 weeks)	0	0	0	0	1	1	15	23	

^a^Compared between fusion mass (score ≥6) and non-fusion mass (score <6)

The lower scores and fusion rates indicated that only a few bones in the fusion masses existed in the control group compared to the treatment group. The typical histologic slides are displayed in Additional file 6: Figure S6, Additional file 7: Figure S7, Additional file 8: Figure S8 and Additional file 9: Figure S9.

## Discussion

In the current study, the efficacy of PEEK device was compared with that of the conventional titanium device in the posterior lumbar fusion surgery with orthotopic paraspinal muscle-pediculated bone flaps in a canine model. Although no significant differences were found upon radiographic evaluation, manual palpation and gross examination at two time points of examination, and histological analysis at 8 weeks, a significant difference was revealed at 16 weeks between the two groups, biomechanically as well as histologically (Additional file 9: Figure S9).

Stress distribution pattern is essential for both biomechanical investigations and remodeling processes. Although similarities in stress distribution pattern between human and canine models have been described [11], there are dissimilarities in walking gestures between dogs and human beings. Hence, the results in dogs may not completely represent the postoperative recovery of the patients. But as the patients were generally placed in supine position for a long time (the lumbar spine is not under stress of long-time upright walking) and the turning over of the patients in bed onto their left- and right-lateral flexion, rotations at the lumbar spine level will be similar to the motions of the lumbar spine of the dogs in walking [11]. Hence, canine model was chosen for the study.

The findings of the present study showed no significant differences in the imaging as well as manual test results between the two groups, which might be due to relatively insufficient subjective evaluations and the classification criteria. Another important reason for the negative results might be the favorable surgical outcomes using the new bone grafting method for posterior lumbar spine fusion, which probably “balanced” the potential differences between the two groups. According to a previous study conducted on the posterior lumbar fusion with orthotopic paraspinal muscle-pediculated bone flaps in a canine model demonstrated increased rate of fusion as well as the quality of fusion mass [12]. Probable reasons behind the improved fusion in the abovementioned model included increased blood supply, better induction of osteogenesis, larger graft bed size and shorter area of fusion, mechanical holding of the bone graft in contact with the nearby bony structure, and prevention of interference of soft tissues with the bone graft. Although the previously conducted animal experiments have demonstrated that this new bone grafting method had superior quality and speed up in the osteogenesis, the non-union still existed. Hence, the current study focused on how to achieve further improvement in the fusion rate and accelerate the osteogenesis [12].

The mechanical holding of the bone graft is an important determinant for the union of the bone graft. For which, two types of devices can be used, either the rigid rod titanium device or the flexible PEEK device [4, 5, 9]. In the current study, it was further explored if PEEK rods can lead to further improvement in the fusion mass by comparing with the conventional titanium rods.

PEEK rods are known to provide mechanical support to the bone graft and remain in contact with the adjacent bony structures. The PEEK rod could generate micro-motion in the region of bone grafting, which further generates the micro-pressure on the osteocytes. Appropriate micro-motion could promote healing of the bones and also accelerates the union of the bone graft (as stated in the Wolff’s law) [4, 5, 9]. The findings of the present study showed that the rate of fusion was high by this method and also confirmed our speculation.

The present study chose PEEK rod with a diameter of 3.0 mm instead of 6.25 mm rod which was routinely used in clinical practices, due to the findings in the previous mechanical studies (data not published). The PEEK rods with diameters of 2.0, 3.0, 4.0, and 5.0 mm were also chosen, and the results showed that the yield load of even the finest PEEK rod (2.0 mm, 197 N) was substantially higher than the lumbar spine stress of the dogs (17.5 N). However, the displacement-deformation under the stress was not significantly different between the 3.0- and 5.0-mm PEEK rods and 3.0-mm titanium rods. Considering the concordance of the diameter with the titanium rod, the present study finally has chosen the 3.0-mm PEEK rod as the union lever. More clinical evidence and mechanical studies are still required to investigate whether the routinely used 6.25-mm PEEK rod could be used instead of a titanium rod.

## Conclusions

In summary, the advantages of this surgical method and the theory of using the elastic rod to generate micro-motion to promote the union of the bone graft have been applied in this study. The micro-motion, stress, and the improved blood supply ensured high quality of bone fusion. According to other studies, the percentage of non-union was about 20% or higher, which was basically associated with the differences in the surgical methods used and the study objects involved in the study [14–18].

The present study has several limitations. For instance, this is an animal study; thus, the results cannot be directly applied in clinical practices. Also, the long-term efficacies have not been investigated in the present study and an additional data to confirm the value of using PEEK rod in clinical practices are still warranted.

Based on the results of this study, we conclude that the elastic fixation by PEEK connecting rod and the elastic micro-motion at the site of fixation could improve the union of the bone graft in the surgery of posterior lumbar spine fusion using the “in situ paraspinal muscle-bone flaps bone grafting method.”

## Additional files

Additional file 1: Figure S1. A. The bone graft bed of the novel posterior spinal fusion model. B. The in situ operation situation of the treatment group (the PEEK rod device and bone graft). C. The in situ operation situation of the control group (the titanium rod device and bone graft). (TIF 3331 kb)
Additional file 2: Figure S2. Schematic diagram of the novel posterior spinal arthrodesis demonstrates the position of split bone and the site of bone graft. (Adapted from [12]). (TIF 912 kb)
Additional file 3: Figure S3. A, B. Radiographs in the treatment group (8 weeks). C, D. Radiographs in the treatment group (16 weeks). E, F. Radiographs in the control group (8 weeks). G, H. Radiographs in the control group (16 weeks). (TIF 1742 kb)
Additional file 4: Figure S4. A, B. Sagittal and coronal 3-dimension CT scan image in the treatment group (8 weeks). C, D. Sagittal and coronal 3-dimension CT scan image in the treatment group (16 weeks). E, F. Sagittal and coronal 3-dimension CT scan image in the control group (8 weeks). G, H. Sagittal and coronal 3-dimension CT scan image in the control group (16 weeks). (TIF 1027 kb)
Additional file 5: Figure S5. A. Photo of the L1–7 specimen in the treatment group (8 weeks). B. Photo of the L1–7 specimen in the treatment group (16 weeks). C. Photo of the L1–7 specimen in the control group (8 weeks). D. Photo of the L1-7 specimen in the control group (16 weeks). (TIF 3807 kb)
Additional file 6: Figure S6. The histologic section of the graft in the control group (8 weeks) in the fusion site (H&E × 10). (TIF 3517 kb)
Additional file 7: Figure S7. The histologic section of the graft in the control group (16 weeks) in the fusion site (H&E × 10). (TIF 3517 kb)
Additional file 8: Figure S8. The histologic section of the graft in the treatment group (8 weeks) in the fusion site (H&E × 10). (TIF 3516 kb)
Additional file 9: Figure S9. The histologic section of the graft in the treatment group (16 weeks) in the fusion site (H&E × 10). (TIF 3512 kb)

## Abbreviations

ASD: Adjacent segment disease
CT: Computed tomography
H&E: Hematoxylin and eosin
PEEK: Polyether ether ketone
PIs: Pars interarticularis
SAPs: Superior articular processes
