# Supplementary figures and images for: A study to compare the efficacy of polyether ether ketone rod device with titanium devices in posterior spinal fusion in a canine model
Source: J Orthop Surg Res. 2017 Mar 9;12:40. doi: 10.1186/s13018-017-0543-x (PMC5345138; doi:10.1186/s13018-017-0543-x)

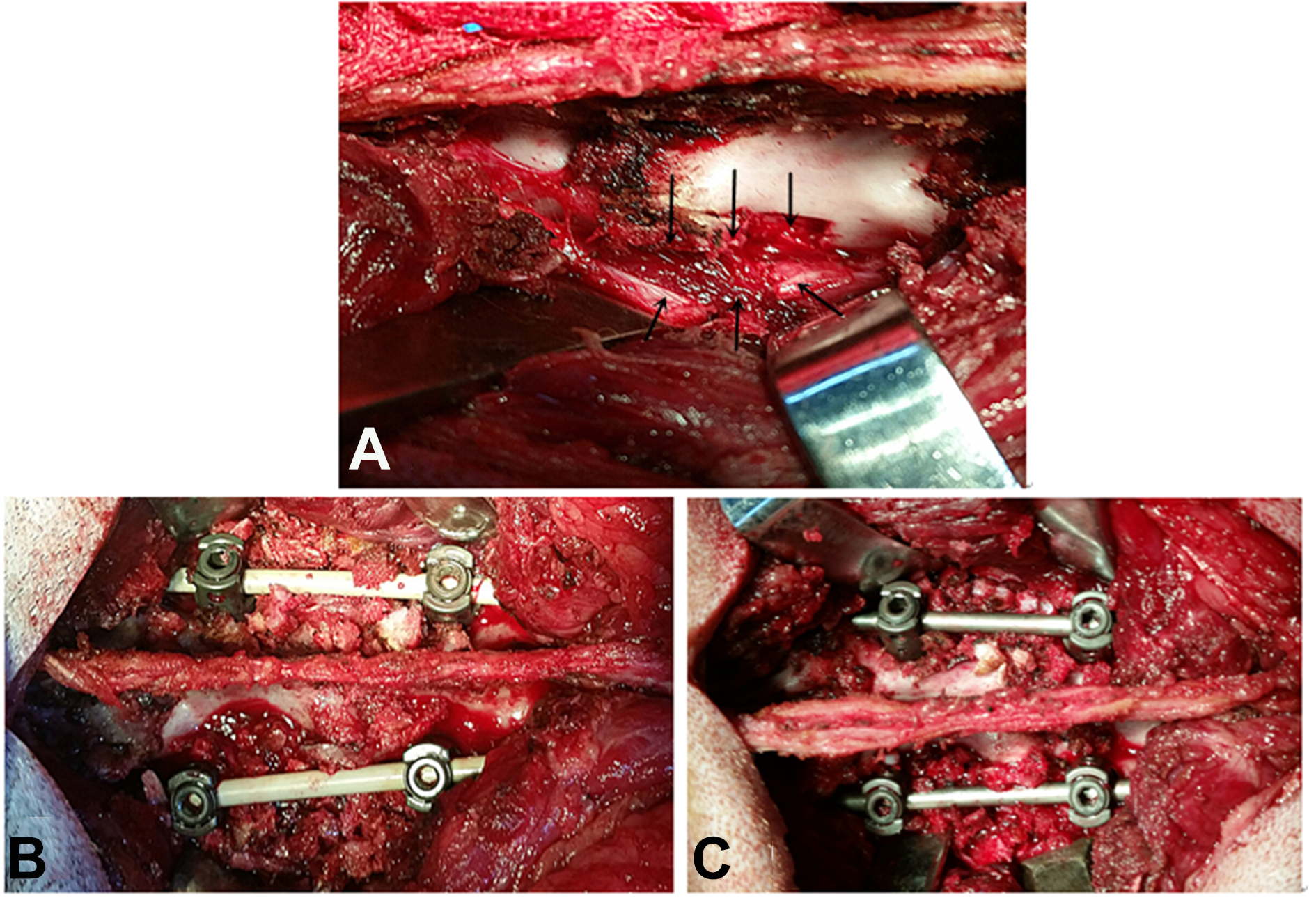

Supplement: Additional file 1: Figure S1. — A. The bone graft bed of the novel posterior spinal fusion model. B. The in situ operation situation of the treatment group (the PEEK rod device and bone graft). C. The in situ operation situation of the control group (the titanium rod device and bone graft). (TIF 3331 kb) [file 13018_2017_543_MOESM1_ESM.tif]

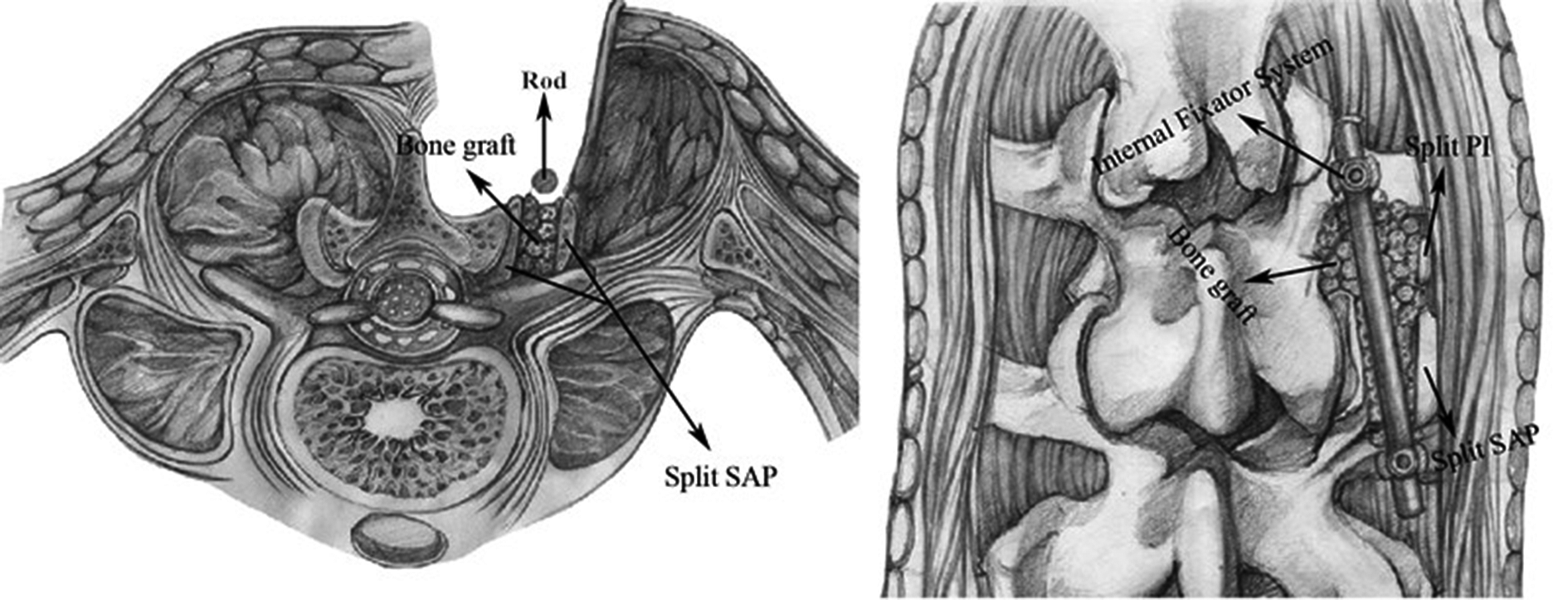

Supplement: Additional file 2: Figure S2. — Schematic diagram of the novel posterior spinal arthrodesis demonstrates the position of split bone and the site of bone graft. (Adapted from [12]). (TIF 912 kb) [file 13018_2017_543_MOESM2_ESM.tif]

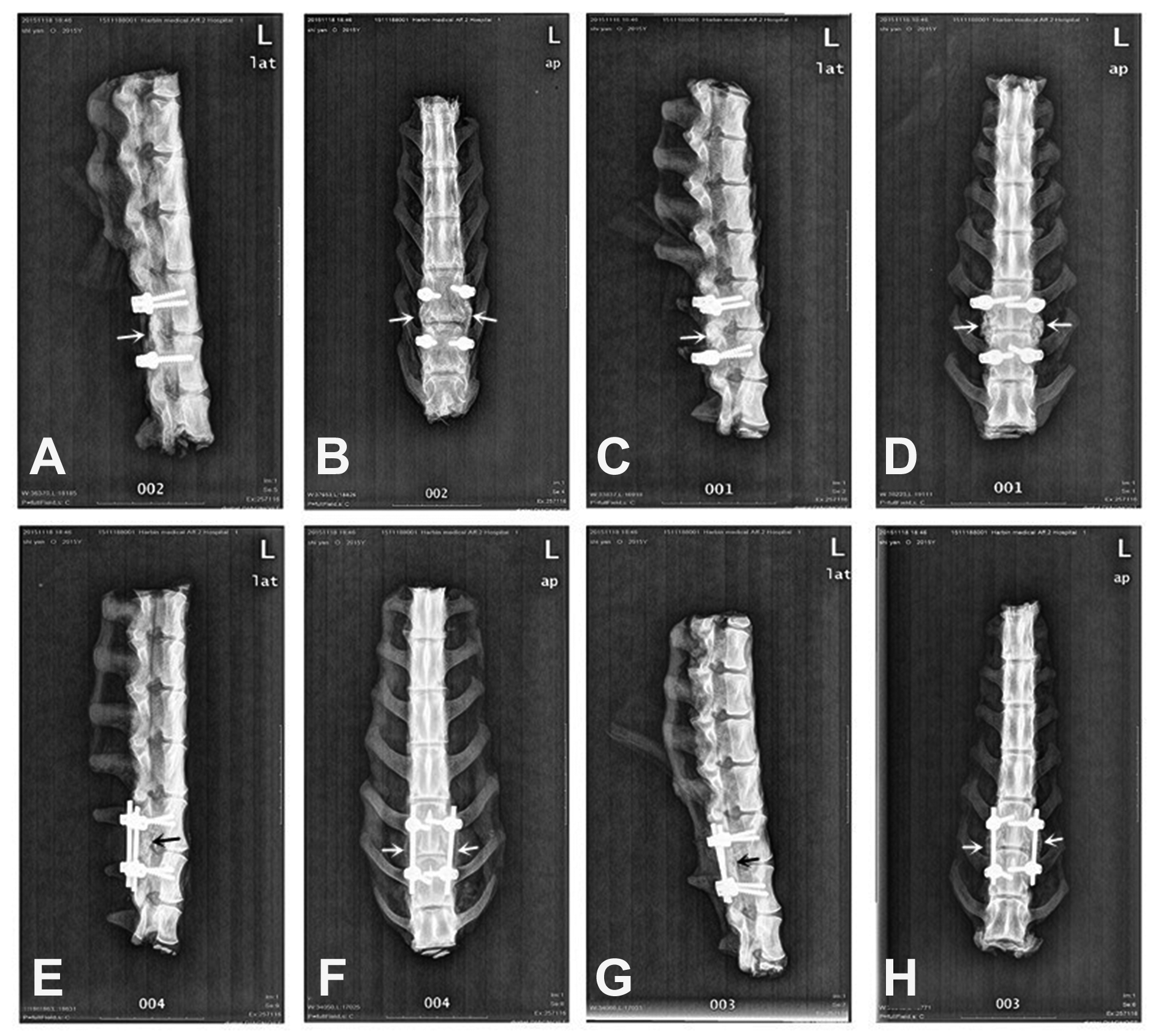

Supplement: Additional file 3: Figure S3. — A, B. Radiographs in the treatment group (8 weeks). C, D. Radiographs in the treatment group (16 weeks). E, F. Radiographs in the control group (8 weeks). G, H. Radiographs in the control group (16 weeks). (TIF 1742 kb) [file 13018_2017_543_MOESM3_ESM.tif]

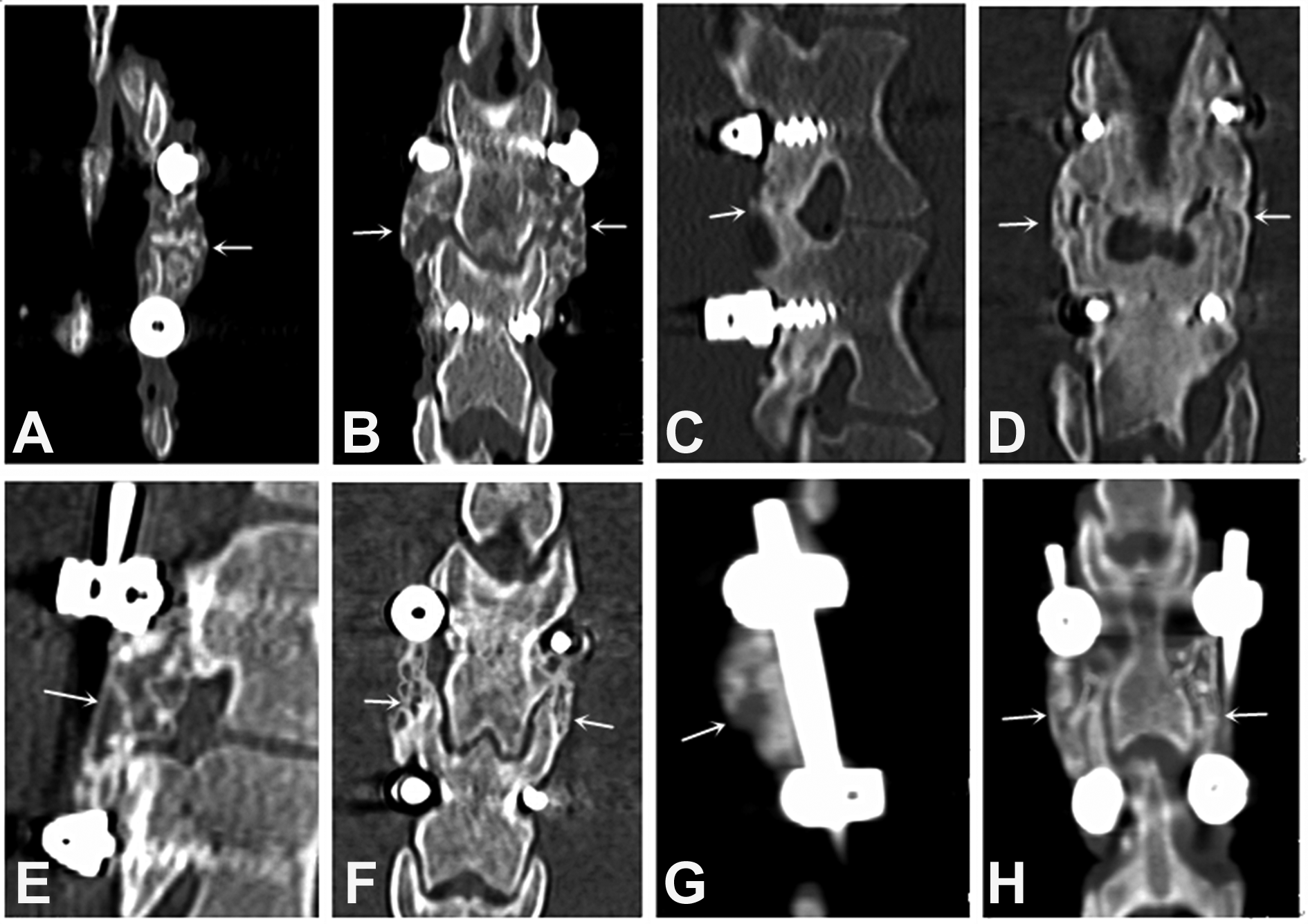

Supplement: Additional file 4: Figure S4. — A, B. Sagittal and coronal 3-dimension CT scan image in the treatment group (8 weeks). C, D. Sagittal and coronal 3-dimension CT scan image in the treatment group (16 weeks). E, F. Sagittal and coronal 3-dimension CT scan image in the control group (8 weeks). G, H. Sagittal and coronal 3-dimension CT scan image in the control group (16 weeks). (TIF 1027 kb) [file 13018_2017_543_MOESM4_ESM.tif]

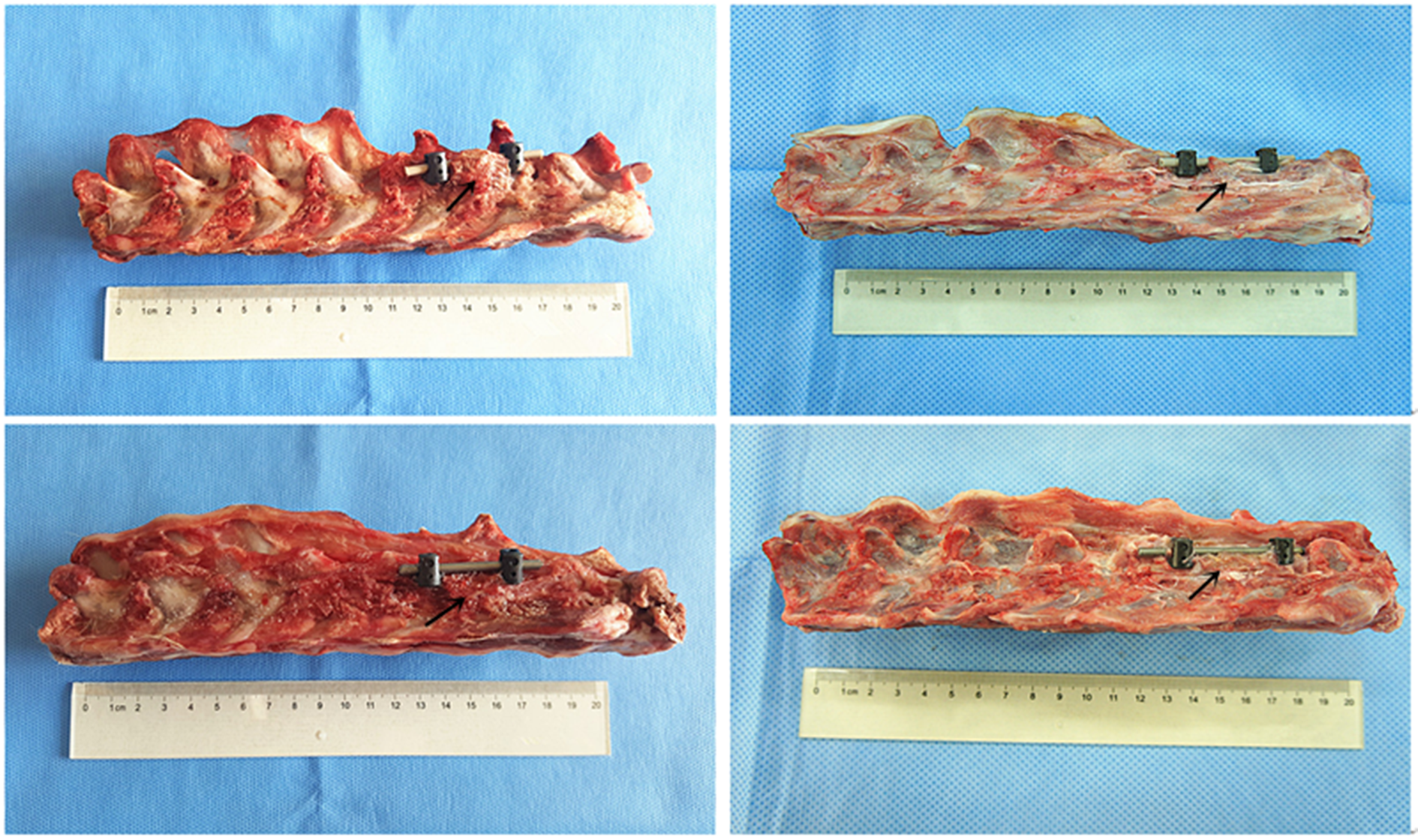

Supplement: Additional file 5: Figure S5. — A. Photo of the L1–7 specimen in the treatment group (8 weeks). B. Photo of the L1–7 specimen in the treatment group (16 weeks). C. Photo of the L1–7 specimen in the control group (8 weeks). D. Photo of the L1-7 specimen in the control group (16 weeks). (TIF 3807 kb) [file 13018_2017_543_MOESM5_ESM.tif]

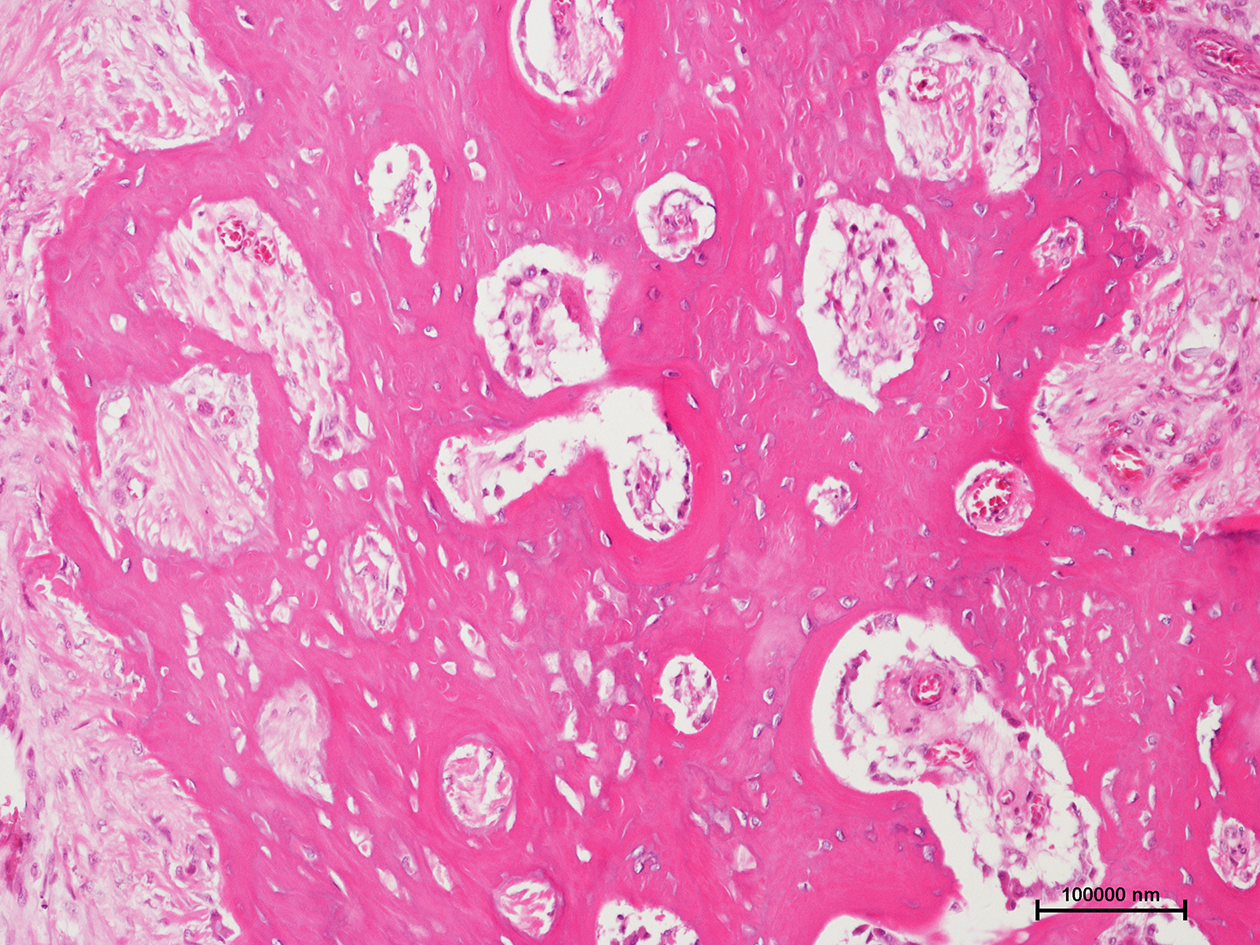

Supplement: Additional file 6: Figure S6. — The histologic section of the graft in the control group (8 weeks) in the fusion site (H&E × 10). (TIF 3517 kb) [file 13018_2017_543_MOESM6_ESM.tif]

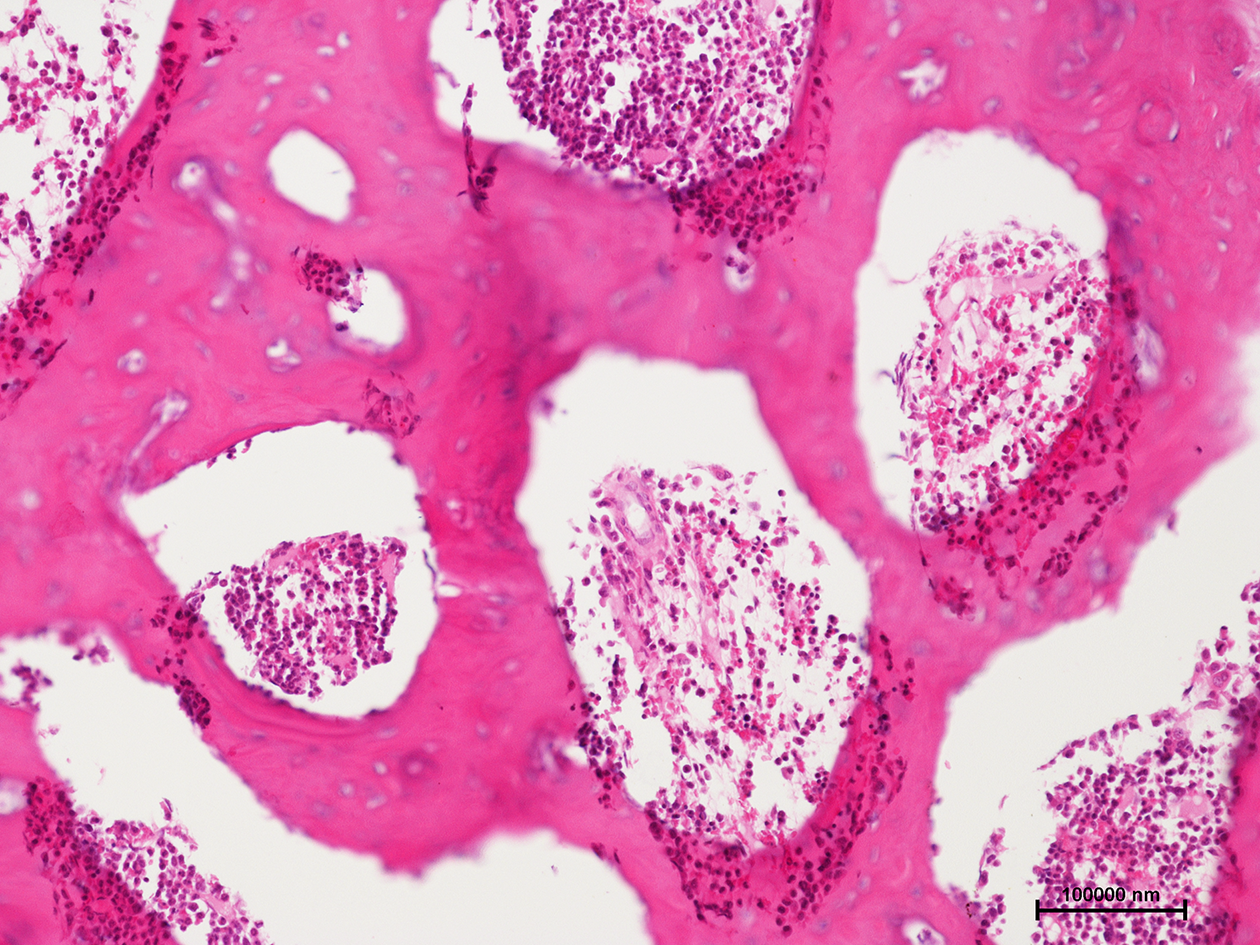

Supplement: Additional file 7: Figure S7. — The histologic section of the graft in the control group (16 weeks) in the fusion site (H&E × 10). (TIF 3517 kb) [file 13018_2017_543_MOESM7_ESM.tif]

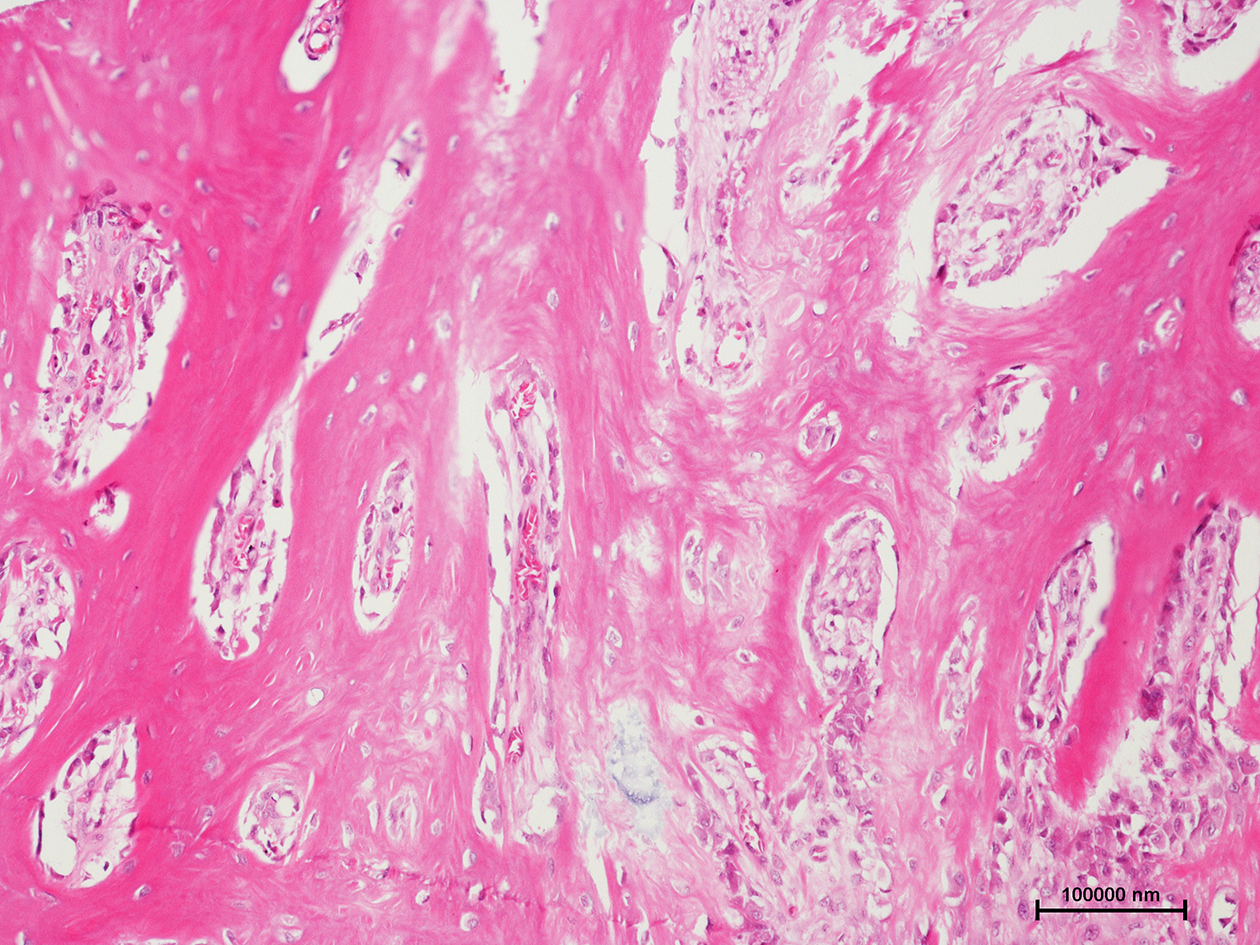

Supplement: Additional file 8: Figure S8. — The histologic section of the graft in the treatment group (8 weeks) in the fusion site (H&E × 10). (TIF 3516 kb) [file 13018_2017_543_MOESM8_ESM.tif]

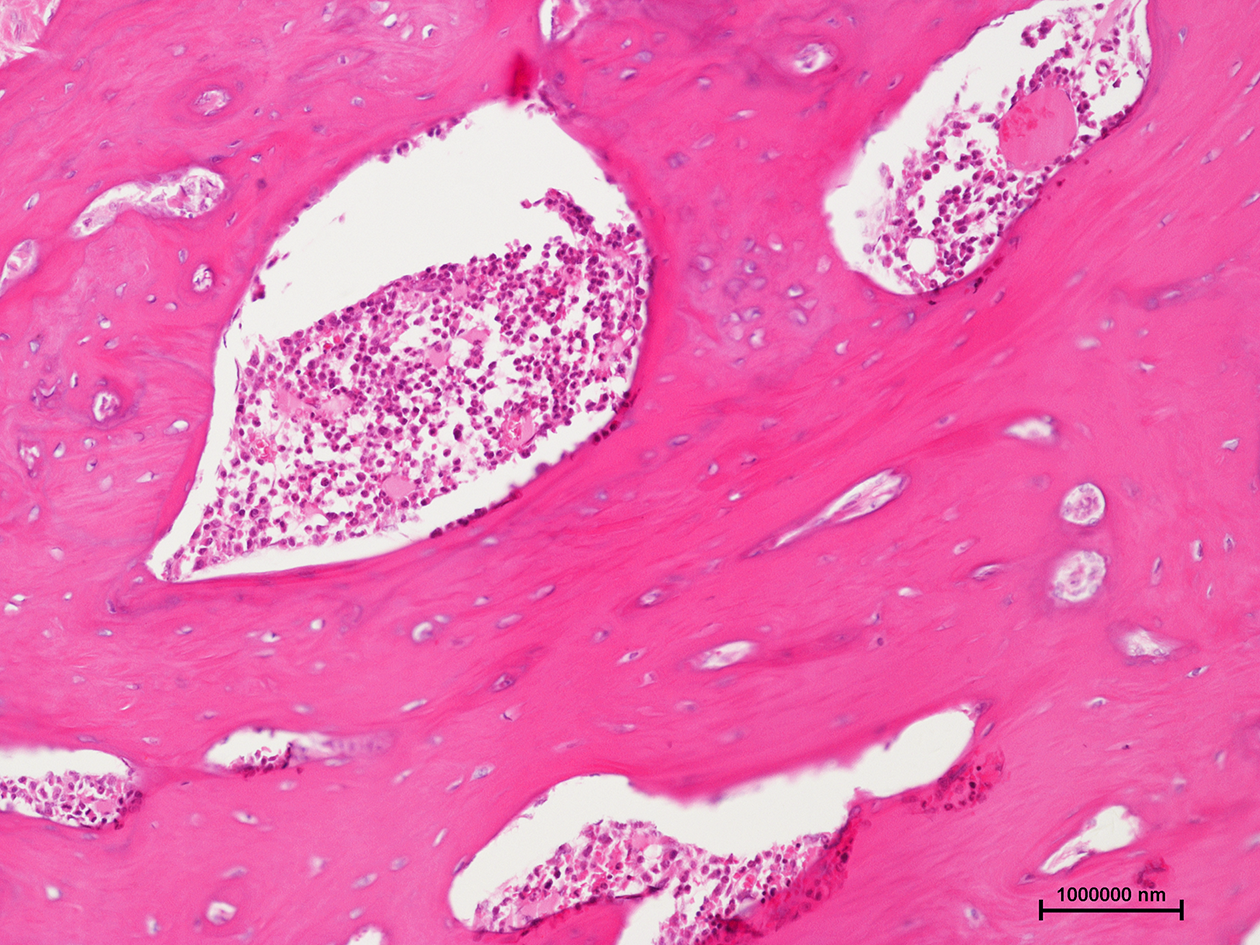

Supplement: Additional file 9: Figure S9. — The histologic section of the graft in the treatment group (16 weeks) in the fusion site (H&E × 10). (TIF 3512 kb) [file 13018_2017_543_MOESM9_ESM.tif]
